# Corrigendum: Number of Childbearing Partners, Status, and the Fertility of Men and Women in the U.S.

**DOI:** 10.3389/fsoc.2020.545366

**Published:** 2021-01-08

**Authors:** Rosemary L. Hopcroft

**Affiliations:** Sociology, University of North Carolina, Charlotte, NC, United States

**Keywords:** sex differences, fertility, evolutionary theory, SIPP data, status

In the original article, there were mistakes in Tables 3 and 4 as published. A human error in software code meant standard errors and significance were reported incorrectly for one variable. The corrected tables appear below [Table 3. Number of childbearing unions by income, net worth, and education and Table 4. Total number of children ever born by income, net worth, education, and number of childbearing unions (only those with at least one childbearing partner)].

**Table 3:** Number of childbearing unions by income, net worth, and education.

	**All cases**			**At least one childbearing partner**		
	**Women**	**Men**	**Difference**	**Women**	**Men**	**Difference**
**Model**	**1**	**2**	**3**	**4**	**5**	**6**
Intercept	0.798^***^ (0.023)	0.241^***^ (0.022)		1.534^***^ (0.019)	1.292^***^ (0.028)	
Age	0.010^***^ (0.000)	0.015^***^ (0.000)	0.005^***^ (0.000)	−0.002^***^ (0.000)	0.000 (0.000)	0.002^***^ (0.000)
Black	0.200^***^ (0.013)	0.170^***^ (0.014)	−0.029 (0.020)	0.191^***^ (0.012)	0.211^***^ (0.015)	0.021 (0.019)
Personal Income ($1,000s)	−0.001 (0.001)	0.003^*^ (0.002)	0.005^**^ (0.002)	−0.002^**^ (0.001)	−0.001^*^ (0.000)	0.002^*^ (0.001)
Personal Net Worth ($1,000,000s)	−0.013 (0.011)	0.001 (0.004)	0.013 (0.013)	−0.011 (0.007)	0.002 (0.003)	0.013 (0.008)
Education (Years)	−0.031^***^ (0.002)	−0.017^***^ (0.002)	0.014^***^ (0.002)	−0.019^***^ (0.001)	−0.012^***^ (0.001)	0.008^***^ (0.002)
N of Cases	29,099	26,182		21,657	16,831	
ADJ. R-square	0.104	0.167		0.043	0.033	

*Unstandardized regression coefficients, standard errors in brackets*.

* *p < 0.05*,

** *p < 0.01*,

*** *p < 0.001*.

**Table 4:** Total number of children ever born by income, net worth, education, and number of childbearing unions (only those with at least one childbearing partner).

	**Women**	**Men**	**Difference**	**Women**	**Men**	**Difference**
**Model**	**1**	**2**	**3**	**4**	**5**	**6**
Intercept	2.650^***^ (0.065)	2.019^***^ (0.052)		1.322^***^ (0.070)	0.560^***^ (0.057)	
Age	0.019^***^ (0.001)	0.021^***^ (0.001)	0.002^**^ (0.001)	0.021^***^ (0.001)	0.021^***^ (0.001)	0.000 (0.001)
Black	0.171^***^ (0.029)	0.231^***^ (0.031)	0.059 (0.039)	0.005 (0.028)	−0.008 (0.026)	−0.013 (0.035)
Personal Income ($1,000s)	−0.011^***^ (0.003)	0.002^**^ (0.001)	0.014^***^ (0.003)	−0.009^***^ (0.003)	0.003^***^ (0.001)	0.012^***^ (0.003)
Personal Net Worth ($1,000,000s)	−0.000 (0.015)	0.005 (0.012)	0.006 (0.020)	0.009 (0.014)	0.003 (0.012)	−0.006 (0.018)
Education (Years)	−0.090^***^ (0.004)	−0.058^***^ (0.004)	0.032^***^ (0.005)	−0.073^***^ (0.004)	−0.045^***^ (0.004)	0.028^***^ (0.004)
Number of child bearing unions				0.870^***^ (0.021)	1.129^***^ (0.025)	0.259^***^ (0.034)
N of Cases	21,657	16,831		21,657	16,831	
ADJ. R-square	0.119	0.088		0.213	0.231	

*Unstandardized regression coefficients, standard errors in brackets*.

* *p < 0.05*,

** *p < 0.01*,

*** *p < 0.001*.

A correction has been made to the abstract; the new text can be seen below:

“Results show that education is always negatively related to the number of child bearing unions and fertility for both men and women. For men, personal income is a positive predictor of both number of child bearing partners and fertility. This is because low income men are more likely to have no child bearing partners at all and not because high income men are more likely to have multiple partners. Both men and women who have a larger number of child bearing partners do have more children, all else being equal, although this effect is stronger for men than for women. Of those with multiple child bearing unions, women with both very high and very low incomes have more children than those with middle incomes.”

The discussion text, paragraph 5, has been updated, the new text can be seen below:

“For both men and women, personal net worth is not a significant predictor of either number of child bearing unions or number of children ever born, despite the fact that the zero order correlations shown in Table 2 showed that personal net worth and personal income are positively associated with both number of child bearing unions and children ever born for men, personal income is negatively associated with number of child bearing unions and children ever born for women, and personal net worth is negatively associated with number of child bearing unions and positively associated with number of children ever born for women. This is because personal income and personal net worth are positively correlated for both men and women (see Table 2) such that high income individuals are also likely to have high personal net worth. This can explain the significant correlations between net worth, number of child bearing unions and number of children ever born seen in Table 2 and the fact that with personal income controlled there is no significant effect of personal net worth on number of child bearing unions (Table 3) and number of children ever born (Table 4) for either men or women.”

The conclusion text, paragraph 3, has been updated, the new text can be seen below:

“The findings when status is measured as education and personal net worth do not support hypotheses from evolutionary biology, as education consistently shows a negative relationship with number of child bearing unions and fertility for men and women, and net worth shows a null relationship with number of child bearing unions and fertility for both men and women when income is controlled.”

The authors apologize for this error and state that this does not change the scientific conclusions of the article in any way. The original article has been updated.

